# Effect of interactive cognitive-motor training on eye-hand coordination and cognitive function in older adults

**DOI:** 10.1186/s12877-019-1029-y

**Published:** 2019-01-28

**Authors:** Pi-Tuan Chan, Wen-Chi Chang, Huei-Ling Chiu, Ching-Chiu Kao, Doresses Liu, Hsin Chu, Kuei-Ru Chou

**Affiliations:** 1 Department of Nursing, En Chu Kong Hospital, Taipei, Taiwan; 2 Department of Nursing, Taipei Veterans General Hospital Yuanshan branches, Yilan, Taiwan; 3 School of Gerontology Health Management, College of Nursing, Taipei Medical University, Taipei, Taiwan; 4 School of Nursing, College of Nursing, Taipei Medical University, No.250, Wu-Hsing Street, Taipei, 110 Taiwan, Republic of China; 5 Department of Nursing, Wan Fang Hospital, Taipei Medical University, Taipei, Taiwan; 6 Institute of Aerospace and Undersea Medicine, School of Medicine, National Defense Medical Center, Taipei, Taiwan; 7 Department of Neurology, Tri-Service General Hospital, National Defense Medical Center, Taipei, Taiwan; 8 Psychiatric Research Center, Taipei Medical University Hospital, Taipei, Taiwan; 9 Department of Nursing, Taipei Medical University-Shuang Ho Hospital, Taipei, Taiwan

**Keywords:** Cognitive-motor training, Eye-hand coordination, Older adults, Cognitive function, Randomized control trial

## Abstract

**Background:**

Poor eye–hand coordination is associated with the symptoms of the early stage of cognitive decline. However, previous research on the eye–hand coordination of older adults without cognitive impairment is scant. Therefore, this study examined the effects of interactive cognitive-motor training on the visual-motor integration, visual perception, and motor coordination sub-abilities of the eye–hand coordination and cognitive function in older adults.

**Methods:**

A double-blind randomized controlled trial was conducted with older adults. Sixty-two older adults were randomly assigned to the experimental (interactive cognitive-motor training) or active control (passive information activity) group, and both groups received 30 min of training each week, three times a week for 8 weeks. The primary outcome was eye–hand coordination, which was further divided into the sub-abilities of visual–motor integration, visual perception, and motor coordination. The secondary outcome was cognitive function. The generalized estimating equation was used to examine differences in immediate posttest, 3-month posttest, and 6-month posttest results between the two groups. Additionally, the baseline effect sizes were compared with the effect sizes of the immediate posttest, 3-month posttest, and 6-month posttests for the experimental group.

**Results:**

There were no statistically significant differences between the intervention and control groups. The only statistically significant difference between the groups was in the attention dimension of cognitive function (*p* = 0.04). The visual–motor integration results showed a small to moderate effect size for pre post comparisons.

**Conclusions:**

The 24 sessions of interactive cognitive-motor training showed no difference to an active control intervention. In the future, this intervention could be further investigated to establish whether it can be superior to an active control group in other populations.

**Trial registration:**

The study protocol has been published on Chinese Clinical Trial Registry (ChiCTR) (registry no.: ChiCTR-IOR-14005490).

**Electronic supplementary material:**

The online version of this article (10.1186/s12877-019-1029-y) contains supplementary material, which is available to authorized users.

## Background

As a result of aging, older adults exhibit a decline in cognitive function. Several risk factors for cognitive impairment have been found, and aging is one of them. Furthermore, cognitive decline due to aging is progressive and irreversible, and it can hamper individuals’ performance of daily activities. Impaired ability to perform activities of daily living (ADL) and instrumental activities of daily living (IADL), which increases individuals’ dependence on others, affects psychological quality of life [1]. Cognitive function comprises a wide range of dimensions, including attention, memory, calculation, orientation, working memory, language, visuospatial ability, problem-solving, executive function, and processing speed [2]. The decline of cognitive functions and the development of dementia increase the probability of disability in older adults, resulting in an economic burden on the public health system [3]. Therefore, a key issue is to identify how cognitive decline in older adults can be delayed and prevent a decline in their ADL ability and eye–hand coordination (EHC). Furthermore, effective interventions that might reduce or delay cognitive decline, or indeed lead to improvements in cognitive function and EHC, are critical for the older adults, especially those at higher risk of cognitive decline.

EHC involves the synergistic functioning of the perception and motor systems [4, 5]. Aging results in physiological changes, and EHC is controlled by the frontal lobe of the cerebrum; cognitive decline affects motor planning and execution and causes problems such as decreased muscular endurance and poor coordination. Thus, cognitive decline greatly affects EHC [6, 7]. As a result of aging, older adults experience a decline in physical activity and a gradual decrease in proprioceptive acuity; the optic nerve axon population also declines, leading to poor EHC [8], which is associated with the symptoms of early stage of cognitive decline [9, 10]. EHC is required for performing daily life activities such as bathing, dressing, exercising, and writing [8]. Severe impairment of EHC affects ADL ability and is closely associated with cognitive decline and nervous system dysfunction. Impaired EHC leads to the decreased ability to perform ADL and social activities in the general population, whereas it may lead to loss of ability for independent living in older adults [11]. Therefore, normal EHC is beneficial toward maintaining the quality of life of individuals. For normal EHC, smooth and natural coordination between the visual and motor systems are essential.

EHC can be divided into the sub-abilities of visual-motor integration (VMI), visual perception (VP), and motor coordination (MC) [12]; these sub-abilities must be in a normal state to ensure smooth and natural EHC. VMI refers to the ability of the visual system to deliver information about the surrounding environment to the brain for organization, analysis, comparison, integration, and other processing. For example, the eyes see the shape of an object, and a pen is used to write it out; this transcribing action is a result of integration between VP and MC. The order of development for VP is pyramid-like and comprises the following dimensions (listed from bottom to top): visual attention, visual scanning, shape identification, visual memory, and visual cognition, which is the highest class. By means of visual input information being processed by perception, these link to cognition and life experiences. MC refers to smoothness, precision, and control during an action. Movement can be divided into coarse action and fine action. Coarse action refers to actions requiring coordination between parts of the body, whereas fine action comprises basic actions of the hand, such as drawing and writing [13].

Interactive cognitive-motor training (ICMT) involves using a computer to simulate a real-world environment; in this training, users receive immediate visual feedback from a projector screen and can experience and interact with the images presented by the computer [14]. Compared with traditional motor training, the advantages of ICMT are as follows: (1) it simultaneously promotes cognitive function: ICMT users are required to simultaneously process information, which enhances their selective attention required to execute tasks, their executive function required to plan or make screen response decisions, and their other promotional abilities required to perform various cognitive functions [15]; (2) its cost and complexity are relatively low, and dropout rates are also lower [16]; (3) it can assist the measurement (using tables), data analysis, and monitoring of the extent of improvement in participants; (4) individual training programs can be provided, and different training stages can be adjusted according to the individual [15]; and (5) it enhances observational learning to provide visual, auditory, and kinesthetic feedback [17].

Studies have indicated that the EHC of older adults can be enhanced through exercises or EHC training [18, 19]. Past theories and empirical studies have further confirmed that interventional measures are effective strategies that improve the cognitive function of older adults [20, 21]. Additionally, empirical studies have indicated that virtual reality training that combines cognition and physical activity is more effective than a training model with either cognition or physical activity [22, 23]. However, studies have primarily focused on interventions in older adults with mild cognitive impairment or dementia, and few empirical studies on the EHC of individuals without cognitive impairment have been conducted. Therefore, this study examined the effects of ICMT on the VMI, VP, and MC sub-abilities of the EHC and cognitive function in older adults. The primary hypothesis of this study is that older adults who completed ICMT would show greater improvements on older adults’ VMI, VP, and MC sub-abilities of EHC when compared with an active control group. The secondary hypothesis of this study is that older adults who completed ICMT would show greater improvements on older adults’ cognitive function when compared with an active control group.

## Methods

### Study design

A double-blind randomized controlled trial was conducted with older adults. Participants received 30 min of ICMT intervention or active control each week, three times a week for 8 weeks, and a total of 24 sessions were conducted. The study protocol has been published on Chinese Clinical Trial Registry (ChiCTR) (registry no.: ChiCTR-IOR-14005490) and approved by the Institutional Review Board of Taipei Medical University (JIRB: 201312037). Our research team conducted series studies. This study focused on the EHC and cognitive function. Previous study focused on the efficacy of gait and balance [16].

### Participants

Participants were primarily older adults who were recruited from senior service centers and community service centers in Northern Taiwan. The inclusion criteria were: (1) age of at least 65 years and ability to communicate in Mandarin Chinese or Taiwanese; (2) no dementia, according to clinical neurological assessment, (Mini-Mental State Examination (MMSE) > 26); [24]; (3) ability to perform ADL independently (after initial inspection), no physical disability, and a perfect score on the Brief Activities of Daily Living assessment; and (4) voluntarily participation and ability to sign the informed consent form. The exclusion criteria were: (1) Alzheimer’s disease diagnosed by physicians or meeting the diagnostic criteria for dementia; (2) severe mental illness or behavior problems and inability to cooperate with the trainer; (3) medical conditions accompanying acute functional decline or cognitive decline (e.g., brain damage); (4) visual impairment or severe cardiovascular diseases resulting in an inability to walk independently; (5) received other cognitive or physical training in the past year; and (6) severe sight, hearing, or communication impairment.

### Randomization and blinding

Using a computer-generated random number table, participants who met the inclusion criteria were randomly assigned to the experimental or active control group by the clinical trial research staff, who had no contact with participants or the researcher. A block randomization design ensured that equal numbers of individuals were assigned to each group. The block size was set at 4 to prevent anyone from guessing the group assignment of the next participant, concealing the group assignment until the end of the study [25]. This study used a double-blind design; thus, neither participants nor researchers were aware of the assigned groups [26]. Participants were only informed that training promotes EHC and cognition. To minimize performance bias, we conducted a block randomization design and an intention-to-treat analysis to estimate the intention-to-treat effect [27]. Group assignment could only be known at the end of data collection, upon checking participant codes.

### Intervention

Participants were randomly assigned to the experimental or active control group. Both groups participated in 30-min training sessions three times a week for a total of 8 weeks (24 sessions in total). In the experimental group, ICMT was performed on the Hot Plus interactive health service system. When using the system, the participants received information visually or acoustically and performed suitable physical responses based on their judgment. Through feedback, coordination between the brain and body can be improved to adjust responses. The entire process challenges the participant’s VP, auditory perception, coordination function, response ability, motor control ability, and cognitive function, and it is suitable not only for cognitive or physical rehabilitation in individuals with impaired cognitive or physical function but also as physical training to maintain physical function in healthy individuals. The ideal exercise model is composed of four interrelated factors, namely, *F*, *I*, *T*, and *T*: *F* stands for exercise frequency, *I* stands for exercise intensity, *T* stands for the time of exercise, and *T* stands for the exercise type, all of which represent the FITT exercise prescription [28]. In this study, ICMT frequency was three times a week for 8 weeks, with 24 sessions in total. Regarding intensity, the Borg Rating of Perceived Exertion Scale (RPE) was 4–7 for the warm-up and 2–4 for the cool-down; the time was 30 min (for each session). The type of ICMT focused on hand coordination exercises, upper- and lower-limb, torso, and functional activity exercises.

The active control group also participated in three sessions of 30-min training per week for 8 weeks, totaling 24 sessions. The active control group used a tablet computer for the passive information activity; training content was divided into reading online newspapers, playing jigsaw puzzles, and finding differences.

### Outcome

This study examined changes in the VMI, VP, and MC sub-abilities of EHC and cognitive function in older adults after ICMT or active control. The primary outcomes were VMI, VP, and MC, which were measured using the Beery–Buktenica Developmental Test of Visual-Motor Integration (Beery VMI) [29]. The secondary outcome was cognitive function. The Montreal Cognitive Assessment (MoCA) was used to assess changes in participants’ cognitive function before and after participation at pretest, posttest, and 3- and 6-month follow-up [30].

#### Primary outcome

##### VMI, VP, and MC of EHC

The Beery VMI was used to assess the VMI, VP, and MC sub-abilities of EHC of participants. This test, which was developed by Beery in 1967, consists of geometric shapes to be reproduced using paper and a pencil, and it includes 27 complete tests for measuring EHC in groups or individuals, with an administration time of 10–15 min. This test includes VMI, VP, and MC tests [29].

1. The VMI test consists of 27 geometric shapes. Participants must copy the geometric shapes on the testing booklet in order, with a total score of 0–27. A higher score indicates higher VMI, and there is no time limit.
2. The VP test requires participants to circle completely similar images among several similar images. It comprises a total of 27 questions, with a total score of 0–27. A higher score indicates higher VP, and the time limit is 3 min.
3. In the MC test, participants must use pencils or pens to trace geometric shapes without crossing the boundaries of the shape. During the test, aids such as a demonstration, starting point of the shape, gray points on the pathway, and double outlines are used to prevent the VP ability from affecting the results. This test comprises a total of 27 questions, with a total score of 0–27. A higher score indicates higher MC, and the time limit is 5 min.

#### Secondary outcome

##### Cognitive function

The MoCA was used to assess the cognitive function of participants. According to previous research, MoCA test better meets the criteria for screening tests for the detection of MCI among patients over 60 years of age than MMSE [30]. Furthermore, the MoCA revealed higher sensitivity to cognitive decline in longitudinal monitoring [31]. In addition to calculating the overall cognitive function score, the scores for the following seven sub items were calculated: visuospatial/executive, naming, attention, language (language fluency), abstraction, delayed recall, and orientation. The testing time was approximately 10–15 min. For participants with less than 12 years of education, 1 point was added to the total score. The highest score is 30, and a higher score indicates higher cognitive function [32]. To distinguish between normal cognitive aging and mild cognitive impairment, the discriminating power of the receiver operating characteristic (ROC) curve was 0.91 (95% confidence interval (CI) = 0.86–1.00). Compared with the area under the curve (AUC) value of MMSE, which was 0.81 (95% CI = 0.72–0.89), the ROC curve had higher discriminating power. For the Taiwanese version used in this study, a cut-off score of 23/24 has been suggested for individuals with mild cognitive impairment, with a sensitivity and specificity of 92 and 78%, respectively [33].

### Sample size

G-Power software was used to calculate the sample size. The F test, MANOVA test, repeated measure test, and between factor tests were employed. The effect size was set at 0.32 [34] in accordance with the randomized results of clinical trials that combined cognition and physical activity, with α = 0.05 and power = 0.9. According to the calculation results, the required sample size was 22 in each group. However, because 3-month and 6-month follow-up were conducted in this study, after the dropout rate was set at 25%, the sample size was set to at least 28.

### Statistical analysis

In this study, SPSS 22.0 software was used for statistical analysis. The chi-square test and independent *t* test were used to verify whether the variables were balanced between the experimental and active control groups after random assignment. To verify the effectiveness of this study, the effect sizes of comparisons between immediate posttest, 3-month posttest, and 6-month posttest results and baseline were calculated for the experimental group. A Cohen’s d value of 0.2 indicates a small effect size, 0.5 indicates a moderate effect size, and 0.8 indicates a large effect size [34, 35]. The generalized estimating equation (GEE) was used to examine differences in immediate posttest, 3-month posttest, and 6-month posttest results between the two groups.

## Results

**Fig. 1:**
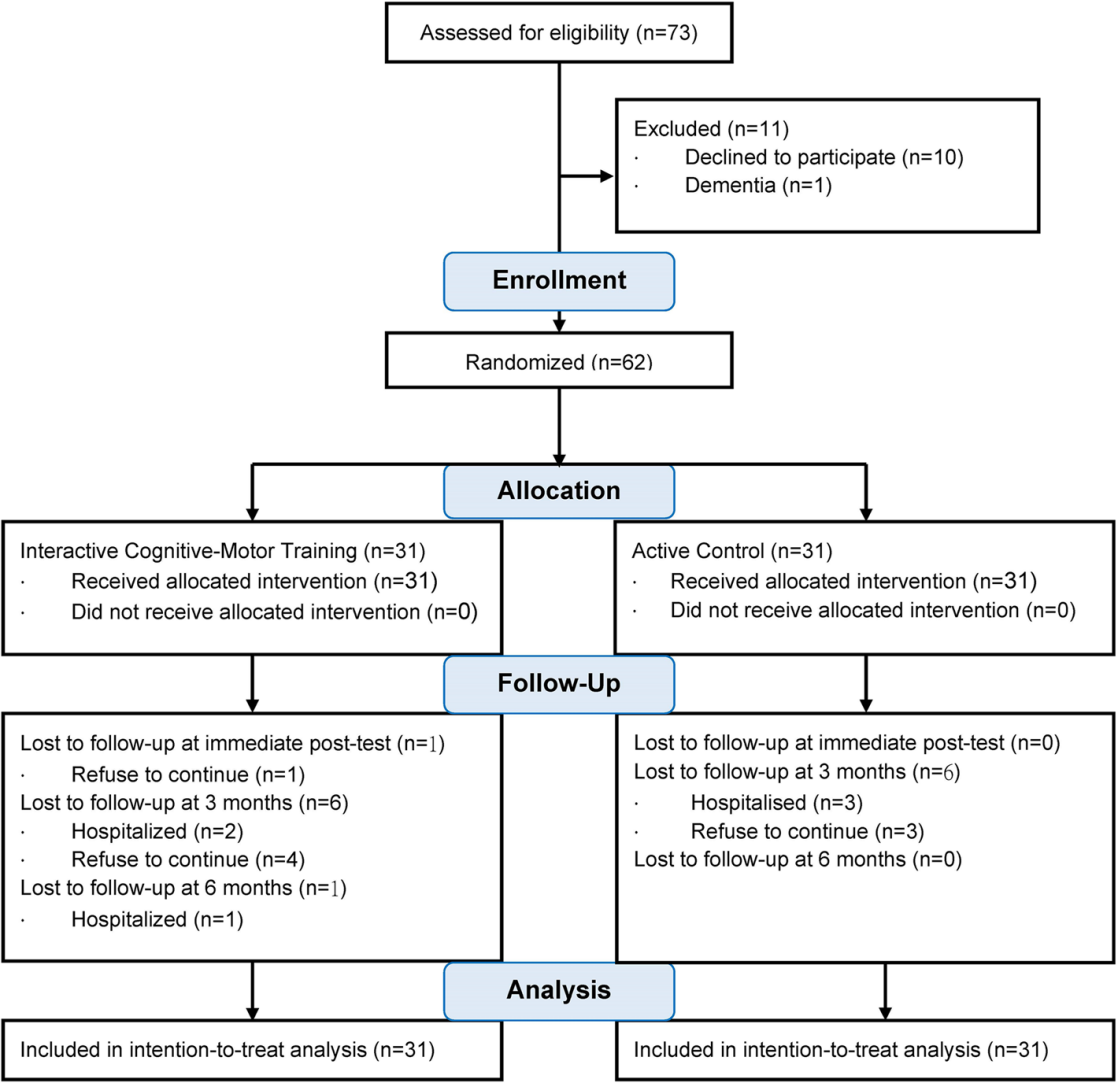
CONSORT 2010 Flow Diagram

A total of 73 participants applied during the recruitment phase of this study. After explanations were provided to them, 10 individuals refused to participate, and one individual had dementia (thereby failing to meet the inclusion criteria). Thus, a total of 62 individuals met the inclusion criteria and signed the informed consent form to participate in the study. After random assignment, the experimental and active control groups each included 31 participants. The detailed recruitment procedure is shown (Fig. 1).

### Participant demographics

This study included 62 participants, of whom 55 were women and 7 were men. The average ages of the experimental and active control groups were 73.52 and 72.32 years, respectively, with no statistically significant difference between the two groups (*P* = .345). The MMSE score for cognitive function was 27.39 ± 1.82 and 27.55 ± 1.99 in the experimental and active control groups, respectively, with no significant difference between the two groups (*P* = .741). The MoCA score was 24.41 ± 3.82 and 24.03 ± 2.52 in the experimental and active control groups, respectively, with no significant difference between the two groups (*P* = .639). The majority of the educational level of the participants was Elementary school and below, with no significant difference between the two groups (*P* = .401) (Additional file 1).

### Eye–hand coordination function

The VMI, VP, and MC sub-abilities of EHC were measured using the Beery VMI.

**Fig. 2:**
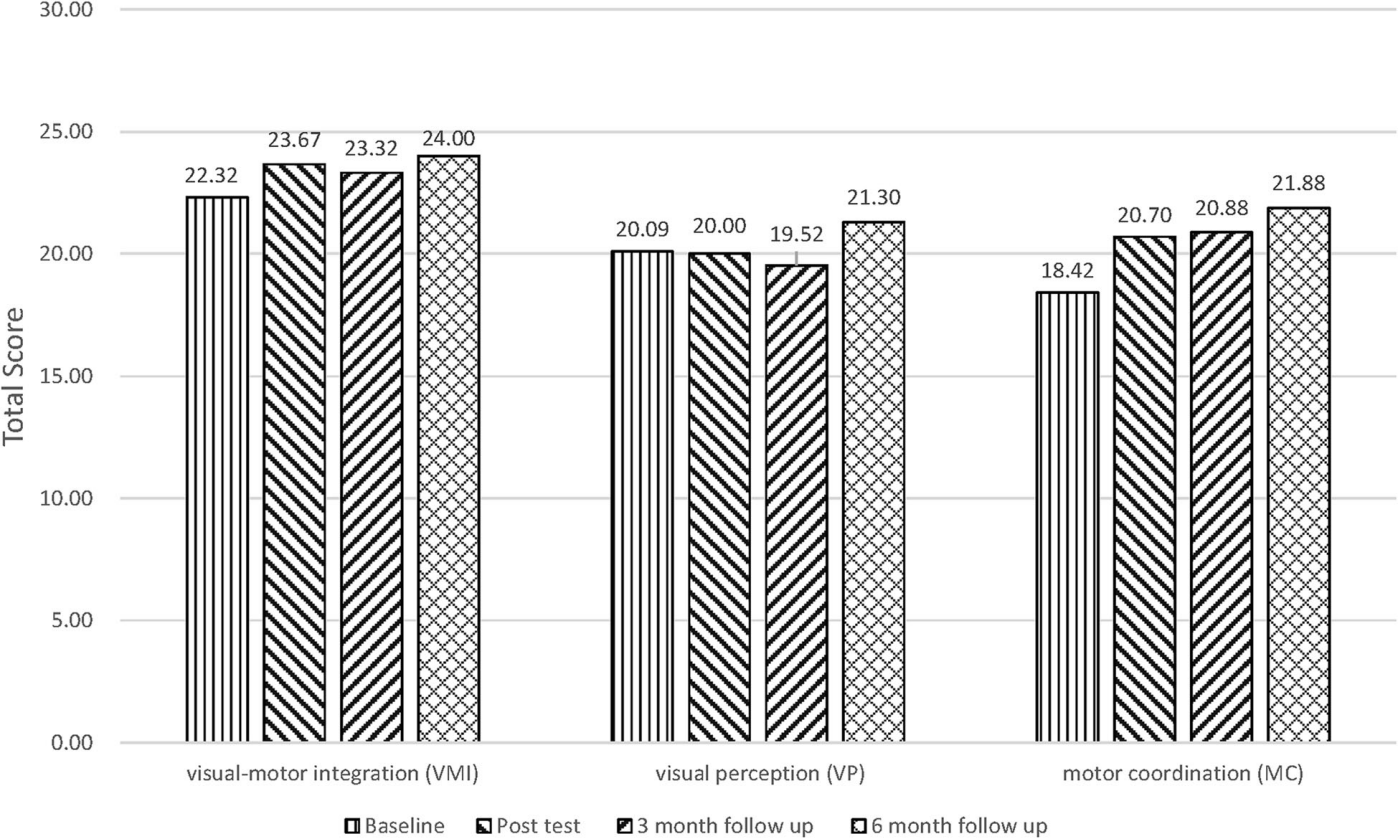
Effects of the visual-motor integration, visual perception, and motor coordination across 4 waves of data collection

1. For the VMI of the experimental group, the effect sizes of 0.50, 0.37, and 0.65 were obtained from the immediate posttest, 3-month posttest, and 6-month posttest results, respectively (by comparing the effect sizes with the baselines). The results indicated that the 24 sessions of ICMT showed a small to moderate effect size for VMI. After control the time effect, the GEE was employed to compare differences between the two groups. The results indicating that the ICMT intervention had no significant effects but maintain the score on VMI between the two groups (*P* > .05) (Fig. 2, Tables 1 and 2).
2. For the VP of the experimental group, the effect sizes of − 0.03, − 0.20, and 0.45 were obtained from the immediate posttest, 3-month posttest, and 6-month posttest results, respectively (by comparing the effect sizes with the baselines). The results indicated that the 24 sessions of ICMT showed a small to moderate effect size for VP. After control the time effect, the GEE was employed to compare differences between the two groups. The results indicating that the ICMT intervention had no significant effects but maintain the score on VP between the two groups (*P* > .05) (Fig. 2, Tables 1 and 2).
3. For the MC of the experimental group, the effect sizes of 0.66, 0.71, and 1.03 were obtained from the immediate posttest, 3-month posttest, and 6-month posttest results, respectively (by comparing the effect sizes with the baselines). The results indicated that the 24 sessions of ICMT showed a moderate to large effect size on MC. After control the time effect, the GEE was employed to compare differences between the two groups. The results indicating that the ICMT intervention had no significant effects but maintain the score on MC between the two groups (*P* > .05) (Fig. 2, Tables 1 and 2).

**Table 1:** Summary of effects on visual-motor integration, visual perception, and motor coordination sub-abilities of eye-hand coordination and global cognitive function

Variables	Interactive cognitive-motor training											Active control group							
	Baseline		Post test			3 month follow up			6 month follow up			Baseline		Post test		3 month follow up		6 month follow up	
	M	SD	M	SD	ES	M	SD	ES	M	SD	ES	M	SD	M	SD	M	SD	M	SD
Berry VMI																			
VMI	22.32	2.97	23.67	2.32	0.50	23.32	2.38	0.37	24.00	2.06	0.65	22.74	3.09	23.66	2.60	23.58	2.47	23.40	2.87
VP	20.09	2.60	20.00	2.92	−0.03	19.52	3.02	−0.20	21.30	2.76	0.45	20.81	3.04	21.63	2.61	21.50	2.47	21.42	3.06
MC	18.42	3.63	20.70	3.29	0.66	20.88	3.27	0.71	21.88	3.08	1.03	19.06	3.97	21.35	3.55	21.39	3.47	22.04	3.07
MoCA																			
Total score	24.41	3.82	24.93	2.72	0.16	25.56	3.18	0.33	25.41	3.61	0.27	24.03	2.53	25.13	2.79	25.96	2.80	26.22	2.85
Visuospatial/Executive	3.74	1.06	3.67	1.03	−0.07	3.80	1.08	0.06	3.92	1.06	0.17	3.80	1.04	3.68	1.08	3.89	0.95	4.11	0.85
Naming	2.26	0.93	2.50	0.78	0.28	2.64	0.81	0.44	2.63	0.82	0.42	2.71	0.59	2.77	0.50	2.82	0.46	2.81	0.62
Attention	5.29	1.04	5.58	0.88	0.30	5.44	0.71	0.17	5.33	0.92	0.04	5.16	0.82	5.33	0.67	5.29	0.94	5.52	0.80
Language	1.81	1.08	2.07	0.83	0.27	2.00	0.96	0.19	1.83	0.96	0.02	2.00	0.89	1.90	0.87	1.86	0.97	1.96	0.80
Abstraction	1.26	0.63	1.33	0.60	0.11	1.20	0.71	−0.09	1.17	0.76	−0.13	1.35	0.66	1.19	0.75	1.46	0.64	1.48	0.58
Delayed recall	2.93	1.60	3.50	1.28	0.39	3.64	1.25	0.49	3.71	1.16	0.56	2.23	1.43	3.26	0.28	3.50	1.26	3.48	1.45
Orientation	5.97	0.18	5.57	0.35	−1.43	5.88	0.33	−0.33	5.91	0.28	−0.25	5.90	0.30	5.90	0.30	5.89	0.31	5.85	0.36

*M* mean, *SD* standard deviation, *ES* Effect size, *Beery VMI* Beery–Buktenica Developmental Test of Visual-Motor Integration, *VMI* Visual-motor integration, *VP* Visual Perception, *MC* Motor Coordination, *MoCA* Montreal Cognitive Assessment

**Table 2:** GEE analysis of effects on visual-motor integration, visual perception, and motor coordination sub-abilities of eye-hand coordination

Variables		B	SE	Wald χ^2^	*P* value
Visual-motor integration (VMI)	Group(EXP)x Time(2nd)^a^	0.43	0.58	0.56	.495
	Group(EXP)x Time(3rd)^a^	0.16	0.67	0.06	.906
	Group(EXP)x Time(4th)^a^	1.02	0.61	2.83	.051
Visual perception (VP)	Group(EXP)x Time(2nd)^a^	−0.92	0.78	1.39	.248
	Group(EXP)x Time(3rd)^a^	−1.27	0.80	2.51	.128
	Group(EXP)x Time(4th)^a^	0.58	0.68	0.75	.390
Motor coordination (MC)	Group(EXP)x Time(2nd)^a^	−0.10	0.89	0.00	.557
	Group(EXP)x Time(3rd)^a^	0.13	0.10	0.02	.853
	Group(EXP)x Time(4th)^a^	0.48	0.95	0.26	.732

“2nd”: the measurement at immediate post-test; “3rd”: the measurement 3 months follow-up; “4th”: the measurement 6 months follow-up

*EXP* Experimental group

^a^Reference group, group (control) × time (baseline)

### Cognitive function

The MoCA was used to assess the cognitive function of the participants. In addition to calculating the overall cognitive function score, the scores for the following seven sub-items related to cognitive function were calculated: visuospatial/executive, naming, attention, language, abstraction, delayed recall, and orientation. After the 24 sessions of ICMT, the overall cognitive function effect sizes of 0.16, 0.06, and 0.27 were obtained from the immediate, 3-month, and 6-month posttest results, respectively. The effect sizes obtained from the immediate, 3-month, and 6-month posttest results for the sub-items are as follows: visuospatial/executive: effect sizes = − 0.07, 0.08, and 0.17; naming: effect sizes = 0.28, 0.44, and 0.42; attention: ES = 0.30, 0.17, and 0.04; language: effect sizes = 0.27, 0.19, and 0.02; abstraction: effect sizes = 0.11, − 0.09, and − 0.13; delayed recall: effect sizes = 0.39, 0.49, and 0.56; and orientation: ES = − 1.43, − 0.33, and − 0.25, respectively (*P* > .05). Among these, the time and group interaction variables of the Attention sub-item were used to compare differences between the two groups. The GEE results revealed statistically significant differences in immediate posttest results between the two groups (*p* = .034), indicating a small effect size (Tables 1 and 3).

**Table 3:** GEE analysis of effects on global and subcomponents of cognitive function

Variables		B	SE	Wald χ^2^	*P* value
Total score of MoCA	Group(EXP)x Time(2nd)^a^	−0.58	0.63	0.86	.243
	Group(EXP)x Time(3rd)^a^	−0.80	0.63	1.56	.164
	Group(EXP)x Time(4th)^a^	−1.19	0.68	3.12	.061
Visuospatial/ Executive	Group(EXP)x Time(2nd)^a^	0.05	0.23	0.05	.803
	Group(EXP)x Time(3rd)^a^	−0.03	0.22	0.02	.832
	Group(EXP)x Time(4th)^a^	−0.13	0.24	0.30	.515
Naming	Group(EXP)x Time(2nd)^a^	0.18	0.15	1.34	.203
	Group(EXP)x Time(3rd)^a^	0.27	0.18	2.27	.143
	Group(EXP)x Time(4th)^a^	0.26	0.20	1.79	.136
Attention	Group(EXP)x Time(2nd)^a^	0.38	0.20	3.37	.034*
	Group(EXP)x Time(3rd)^a^	0.03	0.25	0.01	.981
	Group(EXP)x Time(4th)^a^	−0.31	2.01	0.67	.126
Language	Group(EXP)x Time(2nd)^a^	0.36	0.27	2.29	.165
	Group(EXP)x Time(3rd)^a^	0.34	0.25	1.82	.154
	Group(EXP)x Time(4th)^a^	0.06	0.26	0.06	.798
Abstraction	Group(EXP)x Time(2nd)^a^	0.24	0.18	1.65	.199
	Group(EXP)x Time(3rd)^a^	−0.16	0.19	0.77	.379
	Group(EXP)x Time(4th)^a^	−0.22	0.19	1.32	.250
Delayed recall	Group(EXP)x Time(2nd)^a^	−0.47	0.40	1.38	.211
	Group(EXP)x Time(3rd)^a^	−0.57	0.38	2.30	.134
	Group(EXP)x Time(4th)^a^	−0.48	0.46	1.12	.503
Orientation	Group(EXP)x Time(2nd)^a^	−1.01	0.11	0.90	.345
	Group(EXP)x Time(3rd)^a^	−0.08	0.10	0.59	.386
	Group(EXP)x Time(4th)^a^	0.00	0.10	0.00	.994

“2nd”: the measurement at immediate post-test; “3rd”: the measurement 3 months follow-up; “4th”: the measurement 6 months follow-up

*EXP* Experimental group

*P < .05

^a^Reference group, group (control) × time (baseline)

## Discussion

The findings of this study indicated that 30-min ICMT sessions conducted three times a week for 8 weeks, with a total of 24 sessions, showed a small to large effect size and no significant decline for the VMI, VP, and MC of EHC. Compared with the active control group, the intervention could effectively promote the attention dimension of cognitive function.

EHC plays a crucial role in the successful performance of visual and spatial activities of daily living. According to the principle of motor learning, motor learning includes learning new skills or repeating the same skills, which can enhance visual abilities [36, 37]. The study intervention involved repeating the same motor skill, which may have resulted in the enhancement of visual abilities in older adults, thus further improving their EHC. Additionally, the findings of this study showed that the 24 sessions of ICMT showed an improvement of the score for VMI, VP, and MC of EHC of the experimental group. These findings are consistent with those of previous studies, which have confirmed that poor EHC can be reversed through intervention [18].

The study intervention improved the score of VMI and showed a small to moderate effect size of the experimental group. VMI is the integration of the perceptual and motor system. Previous studies of similar computerized interventions have shown no statistically significant effects on the VMI. Studies have also suggested that visual-motor integration training resulted in no statistically significant decline in the effectiveness on VMI between the two groups in this study [38, 39].

The study intervention improved the score of VP and showed a small effect size in the experimental group. The interventions for VP can be divided into the major categories of developmental, neurophysiologic, or compensatory approaches. According to the theoretical definition, ICMT intervention in this study is a neurophysiologic approach. This type of intervention emphasizes the importance of postural stability to promote the oculomotor efficiency, visual-receptive, and visual-cognitive components in an individual [40]. However, vision damage in older adults, such as presbyopia and cataract, is irreversible and causes a decreased ability to recognize items; thus, this may have resulted in changes in the VP scores of participants.

The study intervention showed moderate to large effect sizes and maintain the score for the MC of the experimental group. When an individual is performing an action, VP involves the use of visual information to confirm the position of each action. During the action, immediate visual or other sensory feedback must be relied on to perform follow-up actions [11]. However, in this study, VP feedback was employed more, and other sensory feedback was employed less, which may have resulted in no statistically significant difference in MC between the two groups. Additionally, during the execution of ICMT, participants were unable to equally use all four limbs for the activity, and they mostly used their dominant hand for the activity, which may have also resulted in maintenance of MC but no statistical difference between the groups.

Although the intervention of the present study was effective for the experimental group, the statistically difference between the experimental and active control groups of VMI, VP and MC could not be obtained. This crucial possible cause may be that the active control group of this study used tablet computers to engage in the passive information activity, and previous studies have indicated the effectiveness of training on tablet computers for MC and VMI [5]. This result is consistent with previous research, which have suggested that tablet computers training can significantly enhance visuospatial abilities, processing speed and memory ability for healthy older adults and patients with mild cognitive impairment [41, 42].

The study intervention showed an immediate, significant, and small effect size for the attention dimension of cognitive function. Attention is the process by which an individual respond to visual or auditory stimuli in the environment, and previous studies have confirmed that computerized training can promote the selective attention of older adults (effect sizes ranged from 0.20 to 1.64) [43, 44], which are consistent with the effect size of this study (Cohen’s *d* = 0.30). Although the study intervention had no statistically significant changes on other sub-items of cognitive function, there was no decreasing trend. In addition, the differences between this study and previous studies may be because ICMT primarily consists of physical activity. The participants were all healthy older adults, their cognitive function was originally high. Thus, the intervention did not result in significant change on other sub-items of cognitive function.

## Conclusions

This study verified that the 24 sessions of ICMT showed small to large effect sizes for the VMI, VP, and MC of EHC and could effectively promote the attention dimension of cognitive function. The advantage of this study is not only that interventional studies were conducted on the EHC of older adults, a topic less frequently discussed in past studies, but also that the VMI, VP, and MC of EHC were analyzed. In addition, because there was no decreasing trend, it may delay the decline of the overall cognitive function of older adults. However, there are few limitations of this study. First, it requires more long-term data, such as data from 1- or 2-year follow-up. Second, the vision damage (such as presbyopia and cataract) in the older adults may cause a decreased ability to recognize the target items of intervention. Finally, a majority of the subjects were female in this is study, which may limit generalizability. Future research should extend the range of participants to older adults with different degrees of cognitive function (such as individuals with mild cognitive impairment or dementia), increase the training session duration, use of a true control group and should conduct long-term follow-up. In clinical practice, because the interactivity of the study intervention was favorable and older adults showed a high participation rate, ICMT should be included in routine community activities to prevent the decline of EHC and cognitive function in older adults.

## Additional file

Additional file 1: Description of participant demographic characteristics (*N* = 62). (DOCX 14 kb)

## Abbreviations

ADL: Activities of daily living
AUC: Area under the curve
Beery VMI: Beery–Buktenica Developmental Test of Visual-Motor Integration
CI: Confidence interval
EHC: Eye–hand coordination
GEE: Generalized estimating equation
IADL: Instrumental activities of daily living
ICMT: Interactive cognitive-motor training
MANOVA: Multivariate analysis of variance
MC: Motor coordination
MMSE: Mini-Mental State Examination
MoCA: Montreal Cognitive Assessment
ROC: Receiver operating characteristic
RPE: Rated perceived exertion
SPSS: Statistical Product and Service Solutions
VMI: Visual-motor integration
VP: Visual perception
