# Correction to: Effect of interactive cognitive-motor training on eye-hand coordination and cognitive function in older adults

**DOI:** 10.1186/s12877-021-02096-y

**Published:** 2021-03-20

**Authors:** Pi-Tuan Chan, Wen-Chi Chang, Huei-Ling Chiu, Ching-Chiu Kao, Doresses Liu, Hsin Chu, Kuei-Ru Chou

**Affiliations:** 1 Department of Nursing, En Chu Kong Hospital, Taipei, Taiwan; 2 Department of Nursing, Taipei Veterans General Hospital Yuanshan branches, Yilan, Taiwan; 3 School of Gerontology Health Management, College of Nursing, Taipei Medical University, Taipei, Taiwan; 4 School of Nursing, College of Nursing, Taipei Medical University, No.250, Wu-Hsing Street, Taipei, 110 Taiwan, Republic of China; 5 Department of Nursing, Wan Fang Hospital, Taipei Medical University, Taipei, Taiwan; 6 Institute of Aerospace and Undersea Medicine, School of Medicine, National Defense Medical Center, Taipei, Taiwan; 7 Department of Neurology, Tri-Service General Hospital, National Defense Medical Center, Taipei, Taiwan; 8 Psychiatric Research Center, Taipei Medical University Hospital, Taipei, Taiwan; 9 Department of Nursing, Taipei Medical University-Shuang Ho Hospital, Taipei, Taiwan

**Correction to: BMC Geriatr 19, 27 (2019)**

**https://doi.org/10.1186/s12877-019-1029-y**

Following publication of the original article [1], the authors would like to make some clarifications in the study.

In the discussion section of our study on page 7, we would like to add these as the first sentences in this section: “Overall, there were no statistical differences between the intervention and control groups except in the attention domain. However, there were outcomes to be noted within the intervention group.” To precede the following sentence: “The findings of this study indicated that 30-min ICMT sessions conducted three times a week … EHC.”

In this article, on page 3, there is a sentence that reads: “The GEE results revealed statistically significant differences in immediate posttest results between the two groups (*p* = .034), indicating a small effect size (Tables 1 and 3).

This sentence should read: “The GEE results revealed statistically significant differences in immediate posttest results between the two groups [for the attention domain] (p = .034), indicating a small effect size (Tables 1 and 3).”

In addition, we would like to offer clarification in the relationships of the following articles and the original study:

Our research team conducted a series of integrated multi-year project that was planned with parallel-group design with multiple interventions, the evaluation of Interactive Cognitive Motor Training (A1) vs. Control (C) on older adults’ different domains of cognitive abilities, visual motor coordination, and gait and balance (used with Hot Plus Interactive Health Service System) [1 & 2], and the evaluation of Executive Function Training (A2) vs. C. on older adults’ different domains of cognitive abilities (used with Rehacom Computer Training Software Executive Function Training module) [3], both sharing the same control (C) group.

The integrated project was complicated, and the measurements of outcome indicators were difficult to digest within the confines of the space. Therefore, in each substudy, distinct aims, different objectives, essential questions, and specific hypotheses were explored further. This study focused on eye-hand coordination (visual-motor integration, visual perception, and motor coordination) and general cognitive function [1]. The other study already mentioned in our article focused on the effectiveness of gait (pace, arm and trunk movement, dynamic stability, and turning) and balance (X-axis and Y-axis direction) [2]. Another study focused on executive function, including mental shifting, working memory, and inhibition [3].

The authors apologize for any confusion these issues may have caused.

References:

[1] Chan, P. T., et al. (2019). Effect of interactive cognitive-motor training on eye-hand coordination and cognitive function in older adults. BMC Geriatrics, 19(1), 27.

[2] Kao, C. C., et al. (2018). Effect of interactive cognitive motor training on gait and balance among older adults: a randomized controlled trial. International Journal of Nursing Studies, 82, 121–128.

[3] Chiu, H. L., et al. (2018). Effectiveness of executive function training on mental set shifting, working memory and inhibition in healthy older adults: A double-blind randomized controlled trials. Journal of Advanced Nursing, 74(5), 1099–1113.
