# Supplementary material for: Effect of interactive cognitive-motor training on eye-hand coordination and cognitive function in older adults
Source: BMC Geriatr. 2019 Jan 28;19:27. doi: 10.1186/s12877-019-1029-y (PMC6350349; doi:10.1186/s12877-019-1029-y)
Supplement: Supplementary file 1 — Description of participant demographic characteristics (N = 62). (DOCX 14 kb) [file 12877_2019_1029_MOESM1_ESM.docx]

Additional file 1. Description of participant demographic characteristics (N=62).

| Variable (category) | | ICMT (n=31) | | AC (n=31) | |  |
| --- | --- | --- | --- | --- | --- | --- |
|  | | Mean (SD) | Number (%) | Mean (SD) | Number (%) | P value |
| Age |  | 73.52 (4.35) |  | 72.32 (5.50) |  | .345 |
| MMSE score |  | 27.39 (1.82) |  | 27.55(1.99) |  | .741 |
| MoCA score |  | 24.41(3.82) |  | 24.03(2.52) |  | .639 |
| Gender |  |  |  |  |  | .229 |
|  | Male |  | 2 (6.5) |  | 5 (16.1) |  |
|  | Female |  | 29 (93.5) |  | 26 (83.9) |  |
| Educational level |  |  |  |  |  | .401 |
|  | Elementary school and below |  | 15 (50.0) |  | 17 (54.8) |  |
|  | Junior high school |  | 3 (8.3) |  | 4 (12.9) |  |
|  | Senior high school |  | 10 (33.3) |  | 5 (16.1) |  |
|  | College and above |  | 3 (8.3) |  | 5 (16.1) |  |
| Marital status |  |  |  |  |  | .965 |
|  | Married |  | 17 (54.8) |  | 18 (58.1) |  |
|  | Divorced |  | 2(6.5) |  | 2 (6.5) |  |
|  | Widowed |  | 12 (38.7) |  | 11 (35.5) |  |
| Religion |  |  |  |  |  | .828 |
|  | Buddhism |  | 15(48.4) |  | 18 (58.1) |  |
|  | Taoism |  | 10 (32.3) |  | 6 (19.4) |  |
|  | Christianity |  | 7 (22.6) |  | 3 (9.7) |  |
|  | No specific religion |  | 4 (12.9) |  | 4 (12.9) |  |
| Occupation |  |  |  |  |  | .307 |
|  | Retired |  | 12 (38.7) |  | 15(48.4) |  |
|  | Unemployed |  | 12 (38.7) |  | 16 (51.6) |  |

*Note:* ICMT, Interactive cognitive-motor training; AC, Active control; SD, standard deviation; MMSE, Mini-mental state examination; MoCA, The Montreal Cognitive Assessment.
